# Peptide Processing Is Critical for T-Cell Memory Inflation and May Be Optimized to Improve Immune Protection by CMV-Based Vaccine Vectors

**DOI:** 10.1371/journal.ppat.1006072

**Published:** 2016-12-15

**Authors:** Iryna Dekhtiarenko, Robert B. Ratts, Renata Blatnik, Lian N. Lee, Sonja Fischer, Lisa Borkner, Jennifer D. Oduro, Thomas F. Marandu, Stephanie Hoppe, Zsolt Ruzsics, Julia K. Sonnemann, Mandana Mansouri, Christine Meyer, Niels A. W. Lemmermann, Rafaela Holtappels, Ramon Arens, Paul Klenerman, Klaus Früh, Matthias J. Reddehase, Angelika B. Riemer, Luka Cicin-Sain

**Affiliations:** 1 Department of Vaccinology, Helmholtz Centre for Infection Research, Braunschweig, Germany; 2 TomegaVax Inc., Portland, Oregon, United States of America; 3 Immunotherapy and prevention, German Cancer Research Center (DKFZ), Heidelberg, Germany; 4 Molecular Vaccine Design, German Center for Infection Research (DZIF), Heidelberg, Germany; 5 Nuffield Department of Medicine, University of Oxford, Oxford, United Kingdom; 6 Dar es Salaam University College of Education, Dar es Salaam, Tanzania; 7 Institute for Virology, University Medical Center Freiburg, Freiburg, Germany; 8 Vaccine and Gene Therapy Institute, Oregon Health and Science University, Beaverton, Oregon, United States of America; 9 Institute for Virology and Research Center for Immunotherapy (FZI), University Medical Center of the Johannes Gutenberg-University Mainz, Mainz, Germany; 10 Department of Immunohematology and Blood Transfusion, Leiden University Medical Center, Leiden, Netherlands; 11 German Center for Infection Research (DZIF), Partner site Hannover/Braunschweig, Germany; 12 Institute for Virology, Medical School Hannover, Germany; Thomas Jefferson University, UNITED STATES

## Abstract

Cytomegalovirus (CMV) elicits long-term T-cell immunity of unparalleled strength, which has allowed the development of highly protective CMV-based vaccine vectors. Counterintuitively, experimental vaccines encoding a single MHC-I restricted epitope offered better immune protection than those expressing entire proteins, including the same epitope. To clarify this conundrum, we generated recombinant murine CMVs (MCMVs) encoding well-characterized MHC-I epitopes at different positions within viral genes and observed strong immune responses and protection against viruses and tumor growth when the epitopes were expressed at the protein C-terminus. We used the *M45*-encoded conventional epitope HGIRNASFI to dissect this phenomenon at the molecular level. A recombinant MCMV expressing HGIRNASFI on the C-terminus of M45, in contrast to wild-type MCMV, enabled peptide processing by the constitutive proteasome, direct antigen presentation, and an inflation of antigen-specific effector memory cells. Consequently, our results indicate that constitutive proteasome processing of antigenic epitopes in latently infected cells is required for robust inflationary responses. This insight allows utilizing the epitope positioning in the design of CMV-based vectors as a novel strategy for enhancing their efficacy.

## Author Summary

Experimental cytomegalovirus (CMV) based vaccine vectors have provided highly encouraging results as innovative vaccine formulations against deadly virus infections, such as Ebola or AIDS. Nevertheless, it has remained incompletely understood why CMV is so efficient at stimulating T-lymphocytes, the immune cells that recognize pathogens within infected cells. We have generated an array of CMV mutants expressing the same antigen in different genes or in different parts of the same gene. This allowed us to identify that the immediate environment of the antigen, rather than properties of the antigen itself, crucially determine the immune protection conferred by CMV-based vaccines, implying that optimal immunity depends on the ability of host cells to degrade CMV proteins into peptides, short units that are recognized by T-cells. Detailed analysis revealed that strong and sustained T-cell immunity occurs only when their antigenic targets are processed by a primitive cellular machinery that is present in all cells of the body, rather than by its newly-evolved counterpart, which is present only in specialized antigen-presenting cells. Most importantly, our results provide a simple strategy to develop improved CMV vaccines by positioning the antigenic peptides at the right spot in CMV proteins.

## Introduction

Cytomegalovirus (CMV) infection maintains the strongest immune response known in clinical medicine, dominating the T-cell memory compartment of seropositive hosts [1]. CMV is a herpesvirus that is never fully eliminated from the host, which may explain why these responses can be detected even at late time points upon initial infection [2, 3]. The T-cell responses to immunodominant CMV antigens appear only to increase with age [4] but at the same time stay functional even in very old, otherwise immunosenescent hosts [5]. Therefore, it has been proposed that CMV recombinants expressing heterologous antigenic determinants [6] may be used as superior vaccine vectors.

Cytomegalovirus (CMV) based vaccine vectors have attracted broad attention over the past years as promising vectors for the induction of protective cellular T-cell responses against a variety of viral, bacterial and tumor targets [6–11]. Rhesus Cytomegalovirus (RhCMV) vectors encoding antigens from the simian immunodeficiency virus (SIV), a virus used in rhesus monkeys as a model of HIV-AIDS disease, sustain a remarkable SIV-specific T-cell response even in CMV-positive animals [11] and clear highly virulent SIV from more than 50% of the monkeys, thus preventing the development of AIDS-like disease [12, 13]. It has been proposed that the ability of CMV-based vaccines to provide this unprecedented level of protection against SIV depends on effector-memory (EM) T-cell responses intercepting viral dissemination at sites of virus entry into the host [14]. How and why CMV sustains this unique immune response is still unresolved and we need to clarify these mechanisms to optimize CMV based vaccines. The cellular and molecular mechanisms of T-cell priming and maintenance by CMV vectors can be addressed in minute detail by infection of inbred and transgenic mice with genetically modified mouse CMV (MCMV).

Experimental MCMV infection was shown to induce persistent infiltrates of CD62L^-^ EM CD8 T-cells in solid organs [15] directed against immunodominant MCMV epitopes [16]. Furthermore, MCMV vectors encoding single antigenic epitopes induced inflationary [17] CD8 T-cell responses against the heterologous epitope, and provided immune control upon challenge with a recombinant vaccinia virus carrying the same epitope [6]. On the other hand, only some epitopes encoded by CMV induce inflationary EM responses [16, 18, 19] and the mechanisms driving this selection are incompletely understood. A plethora of potential mechanisms contributing to inflationary EM responses has been proposed [20], including the efficacy of antigen processing, avidity of peptide binding to MHC molecules and avidity of the T-cell receptor (TCR) binding to peptide-MHC-I (pMHC-I) complex. We showed previously that inflationary responses depend on the context of epitope expression, rather than peptide-intrinsic properties [8]. On the other hand, the viral gene *M102* was shown to simultaneously induce inflationary and non-inflationary CD8 T-cell responses [18]. Therefore, promoter activity alone could not explain the entire selection process.

We show here that C-terminal localization of a peptide results in drastically improved immune protection by CMV-based vaccine vectors. We also show that an MCMV peptide that induces conventional CD8 T-cell responses from its native site becomes inflationary when transferred on the C-terminus of the same viral protein. Finally, we show that these striking differences in size and type of response correspond to the presence of the pMHC-I complexes on the surface of *in vitro* virus-infected endothelial cells and that the effect critically depends on the availability of the peptide for constitutive proteasomal processing.

## Results

### C-terminal localization provides optimal immune protection

**Fig 1:**
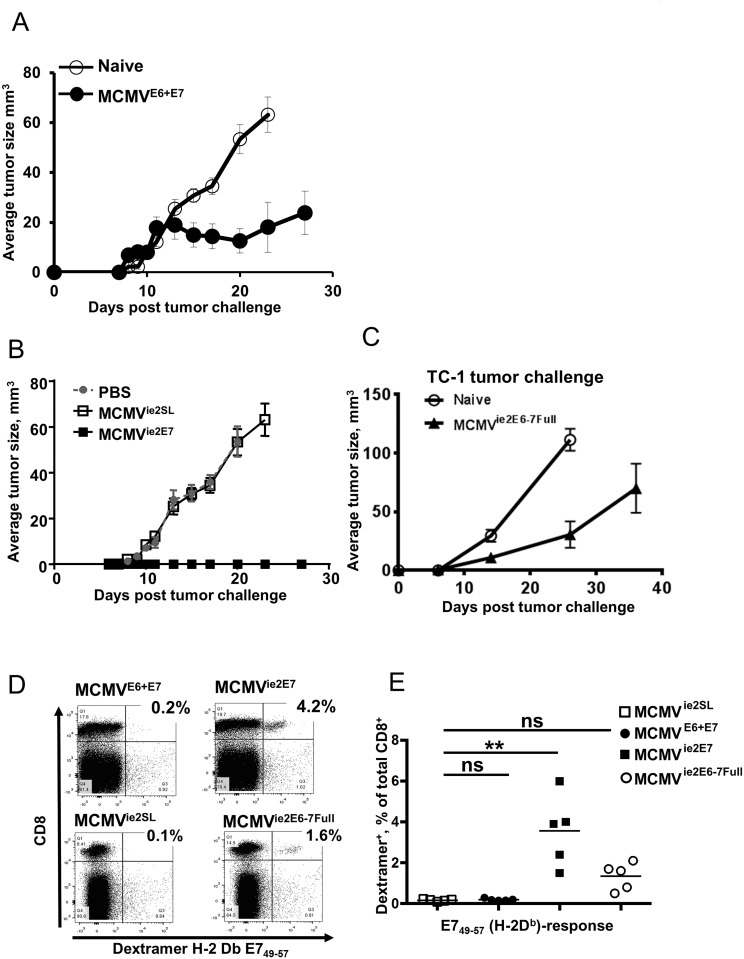
Immune protection by C-terminal epitope localization in a CMV vaccine vector. (**A-C**) Mice were prime/boosted at 4 weeks intervals with recombinant MCMVs or control virus and challenged with 2.5x10^4^ TC-1 cells/mouse at least 10 weeks after priming. Tumor size measured by caliper (mean +/- SEM is shown). **(A)** Immunization was performed with 10^6^ PFU of MCMV^E6+E7^ (n = 9) and tumor growth compared to unvaccinated (naïve) controls (n = 10) (**B**) Mice were immunized with 10^5^ PFU of MCMV^ie2E7^, and compared to MCMV^ie2SL^ or PBS control (n = 10 in each group) (**C**) Mice were prime/boosted with 10^5^ PFU of MCMV^ieE6-7Full^ (n = 9) and compared to unvaccinated controls (n = 12) (**D**) Representative flow cytometry plots of dextramer-stained blood lymphocytes from mice infected with 10^5^ PFU of MCMV^E6+E7^, MCMV^ie2E7^, MCMV^ie2E6-7Full^ or MCMV^ie2SL^ and analyzed by D(b) E7_49-57_ dextramer staining for the presence of E7-specific CD8 T cells at 21 weeks post-priming. (**E**) Group values from dextramer staining as in panel D are shown (each symbol is a mouse; horizontal line shows the median). Significance was assessed by Kruskal—Wallis test followed by Dunn’s post hoc analysis for indicated columns. **p < 0.01, ns—not significant.

To test immune protection by CMV-based vectors in a model of human papilloma virus (HPV) induced cancer, we generated a recombinant MCMV (MCMV^E6+E7^) expressing the full-length E6 and E7 proteins of the HPV strain 16 (HPV16). We placed E6 and E7 under the control of the HCMV immediate-early (IE) promoter, because we showed previously that an IE promoter induces stronger CD8 T-cell responses than an early one [8]. Moreover, we utilized an MCMV backbone lacking the viral genes *m1* to *m16* [21] and thus providing ample cloning capacity. At 10 weeks post infection (p.i.) with MCMV^E6+E7^, the response to the immunodominant D^b^-restricted E7_49-57_ peptide RAHYNIVTF was detectable, although weaker than the response to the endogenous inflationary IE3 epitope (S1 Fig). We tested next the immune protection by MCMV^E6+E7^ against a challenge with E6+E7 transformed TC-1 tumor cells [22]. 25,000 TC-1 cells were administered at 27 weeks post immunization and MCMV^E6+E7^ immunized mice showed reduced tumor growth in comparison to mock-vaccinated mice, but not a complete block of tumor growth (Fig 1A). To define if protection was mediated by CD8 T-cell responses, we generated another MCMV recombinant expressing only the MHC class I epitope E7_49-57_ on the C-terminus of the *ie2* protein (MCMV^ie2E7^). Control mice were immunized with the recombinant MCMV^ie2SL^, expressing the SSIEFARL epitope in the same location [8], or mock-immunized with PBS. Tumor growth upon challenge with TC-1 cells was completely prevented in MCMV^ie2E7^ (Fig 1B), and the mice remained tumor-free for the duration of the experiment. The improved immune protection by MCMV^ie2E7^ over MCMV^E6+E7^ was unexpected, because MCMV^E6+E7^ expressed numerous antigenic epitopes and MCMV^ie2E7^ only one, which was also present in MCMV^E6+E7^. We reasoned that improved protection by MCMV^ie2E7^ could have been due to differences in promoter activity between HCMV *IE* and MCMV *ie2*, or by the better priming due to the C-terminal peptide localization. We analyzed this by generating a third MCMV mutant, where we fused the full-length E6 and E7 proteins to the C-terminus of the ie2 protein of a full-length MCMV (MCMV^ie2E6-7full^). Hence, MCMV^ie2E6-7full^ used the MCMV *ie2* promoter to drive the full-length E6+E7 transcriptional unit, but RAHYNIVTF was located in its native site, and not at the C-terminus. Six out of 9 mice vaccinated with MCMV^ie2E6-E7full^ and challenged with 25,000 TC-1 cells displayed tumor growth (Fig 1C), arguing that epitope localization, rather than promoter activity, resulted in absolute immune protection by MCMV^ie2E7^. In that case, CD8 T-cell responses against the E7_49-57_ epitope RAHYNIVTF should be stronger in MCMV^ie2E7^ infection than in MCMV recombinants expressing the full length protein. We compared immune response by pMHC-I dextramer staining (representative dot blots in Fig 1D), and the response was undetectable in MCMV^E6+E7^ infected mice, stronger in the MCMV^ie2E6-7full^ group and strongest upon MCMV^ie2E7^ infection (Fig 1E). In sum, our data argued that C-terminal epitope localization improved CD8 T-cell responses and thus, immune protection.

### C-terminal epitope localization drives protective effector memory CD8 T-cell responses

**Fig 2:**
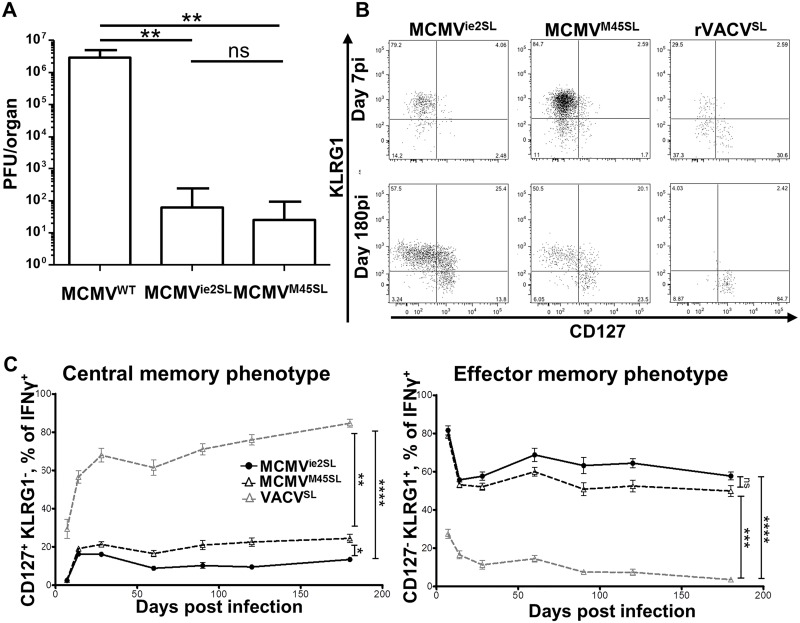
Peptide C-terminal localization results in better protection and induction of effector memory CD8 T-cell response. **(A**) 129Sv female mice were i.p. infected with 2x10^5^ PFU MCMV^WT^, MCMV^ie2SL^ or MCMV^M45SL^ (n = 10 in each group) and 8 months later challenged with 10^6^ PFU of VACV^SL^. Seven days post challenge, ovaries were titrated for infectious vaccinia by plaque assay. Histograms show group means, error bars are standard deviations. Significance was assessed by Kruskal—Wallis test followed by Dunn’s post hoc analysis for indicated columns. **p < 0.01, ns—not significant. **(B-C)** 129/Sv mice were infected intraperitoneally (i.p.) with 2x10^5^ PFU of MCMV^ie2SL^, MCMV^M45SL^ or 10^6^ PFU of VACV^SL^. Blood leukocytes were stimulated with the SSIEFARL peptide at 7, 14, 28, 60, 90, 120, 180 dpi. Cells were surface-stained for CD3, CD4, CD8, CD11a, CD44, KLRG1, CD127 and intracellularly for IFNγ expression and analyzed by flow cytometry. (**B**) Representative dot plots of KLRG1 and CD127 expression in IFNγ producing cells upon 6h SSIEFARL in vitro re-stimulation on days 7 and 180 p.i‥ (**C**) Left graph—epitope specific cells with the CM phenotype (CD127^+^KLRG1^-^). Right graph—epitope specific cells with the EM phenotype (CD127^-^KLRG1^+^). The experiment was performed three times independently, at 5 mice per group in each experiment, and grouped averages +/- SEM from all three experiments are shown. Significance on day 180 p.i. was assessed by Kruskal—Wallis test followed by Dunn’s post hoc analysis. *p<0.05, **p < 0.01, ***p < 0.001, ****p < 0.0001, ns—not significant.

To test if immune protection by a C-terminally expressed epitope would be generally applicable to another epitope and another MCMV gene, we used our previously described MCMVs expressing the K^b^-restricted SSIEFARL epitope as a C-terminal tag on the ie2 (MCMV^ie2SL^) or on the M45 (MCMV^M45SL^) protein [8]. Mice were immunized with either of the mutants or wild-type MCMV as control and challenged with a recombinant vaccinia virus expressing the same epitope (rVACV^SL^). Both MCMVs expressing the SSIEFARL epitope significantly controlled rVACV^SL^ replication (Fig 2A), providing further evidence that C-terminal localization of the antigenic epitope will result in immune protection. It has been proposed that the immune protection induced by CMV-based vaccine vectors rests on the induction of antigen-specific EM T-cells [12]. Hence, we analyzed the phenotype of SSIEFARL-specific CD8 T cells upon MCMV^ie2SL^, MCMV^M45SL^ or rVACV^SL^ infection. Blood leukocytes were *in vitro* restimulated with the peptide for 6h and IFNγ responding cells were classified according to CD127 and KLRG1 expression into EM (KLRG1^+^CD127^-^) or central memory (CM) (KLRG1^-^CD127^+^) cells. All infections induced a predominantly EM phenotype in IFNγ^+^ cells at 7 days post infection (dpi), but this response remained EM in both MCMV infections until 180 dpi (Fig 2B and 2C), while it rapidly shifted to a CM phenotype in rVACV infection (Fig 2C).

### The surface phenotype of CD8 T cells recognizing MCMV antigens is not determined by the gene expression pattern

**Fig 3:**
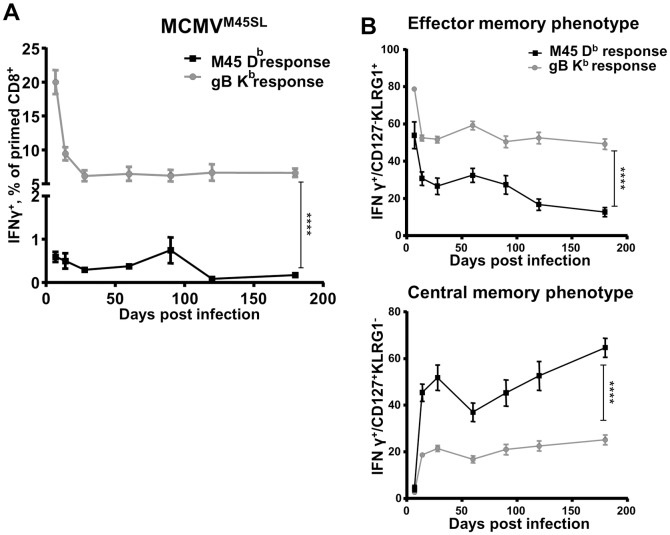
Gene expression context does not define the quality of CD8 responses to MHC-I restricted epitopes of MCMV. 129/Sv mice were infected intraperitoneally (i.p.) with 2x10^5^ PFU of MCMV^M45SL^. Blood leukocytes were stimulated with the SSIEFARL or the HGIRNASFI peptide at 7, 14, 28, 60, 90, 120, 180 dpi. Cells were surface-stained for CD3, CD4, CD8, CD11a, CD44, KLRG1, CD127 and intracellularly for IFNγ expression and analyzed by flow cytometry. (**A**) Cells responding to the SSIEFARL (gB K^b^ response) or the HGIRNASFI (M45D^b^ response) peptide. (**B**) Upper graph—epitope specific cells with the EM phenotype (CD127^-^KLRG1^+^). Lower graph—epitope specific cells with the CM phenotype (CD127^+^KLRG1^-^). The experiment was performed three times independently, at 5 mice per group in each experiment, and grouped averages +/- SEM from all three experiments are shown. Significance on day 180 p.i. was assessed by a Mann—Whitney *U* test. ****p < 0.0001.

Since the *M45* gene encodes a natural D^b^-restricted epitope (HGIRNASFI), which induces non-inflationary CM responses [18, 23, 24], we were surprised by the EM response to SSIEFARL upon MCMV^M45SL^ infection (Fig 2). We showed previously that MCMV^ie2SL^ induces inflationary CD8 T-cell responses to SSIEFARL, whereas the responses contract by 14 dpi in MCMV^M45SL^ infection [8]. We considered that the C-terminal modification of the M45 protein could have destabilized the whole protein and altered the phenotype of the responses against all epitopes encoded by this *M45* variant. Therefore, we compared the CD8 T-cell response to SSIEFARL and HGIRNASFI epitopes upon MCMV^M45SL^ infection and noticed a drastic difference in their size and the quality. SSIEFARL induced ~30-fold stronger responses than HGIRNASFI at all times p.i. (Fig 3A) and cells responding to HGIRNASFI showed a CM phenotype (CD127^+^, KLRG1^-^) within weeks p.i., while SSIEFARL-specific cells retained an EM (CD127^-^, KLRG1^+^) phenotype for up to 180 dpi (Fig 3B). Similarly, CD62L expression remained low on SSIEFARL specific cells, regardless of expression context (S1B Fig). Since the C-terminal modification did not alter the phenotype of responses against HGIRNASFI, the EM phenotype of responding T cells was specific for SSIEFARL.

### C-terminal peptide localization results in effector memory CD8 T-cell responses

The test if the difference in responses to SSIEFARL and HGIRNASFI depended on peptide sequence or localization within the protein, we generated a new recombinant MCMV, where the HGIRNASFI epitope was moved from its original location to the M45 C-terminus. First we generated a negative-control virus lacking the HGIRNASFI epitope (MCMV^M45I->A^), by replacing the D^b^-anchoring isoleucine at the HGIRNASF**I** C-terminus with an alanine (S2A Fig), which precluded efficient peptide processing and anchoring to the D^b^ molecule (the approach is described in [25]). On this background we introduced the HGIRNASFI peptide at the C-terminal end of the M45 protein (S2B Fig). The new recombinant virus (MCMV^M45Cterm^) showed no growth defects *in vitro* and *in vivo* (S2C and S2D Fig).

**Fig 4:**
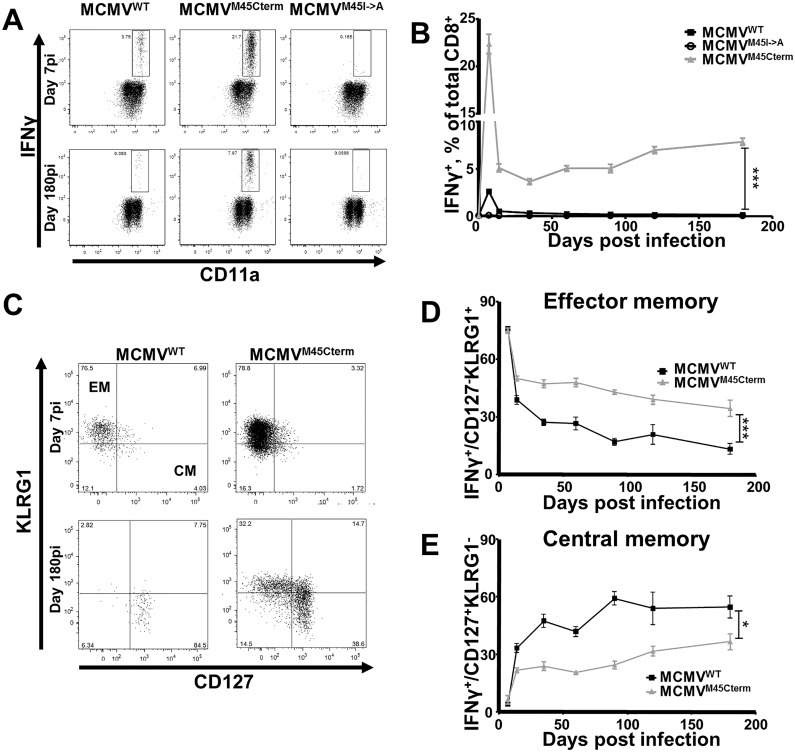
Epitope localization defines the quality of CD8 T-cell responses. (**A**) Representative dot plots of intracellular IFNγ expression at 7 and 180 dpi upon HGIRNASFI peptide stimulation. (**B**) Grouped means +/- SEM of cells responding to the HGIRNASFI peptide at 7, 14, 28, 60, 90, 120, 180 dpi. (**C**) Representative dot plots of the surface expression of CD127 and KLRG1 on HGIRNASFI specific CD8 T cells at 7 and 180 dpi with MCMV^WT^ or MCMV^M45Cterm^. The staining was used to define the CM (CD127^+^KLRG1^-^) and the EM (CD127^-^KLRG1^+^) subsets. (**D, E**) Grouped means +/- SEM of the percentage of EM (**D**) or CM (**E**) cells in the HGIRNASFI-responding subset at indicated time points p.i‥ The experiment was performed twice, at 5 mice per group in each experiment, and pooled results are shown. Significance on day 180 p.i. was assessed by a Mann—Whitney *U* test. *p<0.05, ***p < 0.001.

Mice were infected with either of the new mutant viruses or with MCMV^WT^ and HGIRNASFI-specific CD8 T-cell frequencies were monitored over 180 days by peptide restimulation of blood leukocytes and intracellular staining for IFNγ. As expected, HGIRNASFI-specific CD8 T cells were undetectable in mice infected with the MCMV^M45I->A^ recombinant, but clearly responded to MCMV^WT^ infection. Remarkably, MCMV^M45Cterm^ elicited an ~ 8-fold stronger response to HGIRNASFI at 7 dpi than MCMV^WT^ (Fig 4A, **upper row**). While HGIRNASFI-specific CD8 T cells declined from this peak in both groups by 180 dpi, their percentage was about 70-fold higher in MCMV^M45Cterm^ than in MCMV^WT^ infected mice (Fig 4A, **bottom row**). Detailed analysis of the response kinetics showed that responses upon MCMV^WT^ infection contracted by 14 dpi and remained low thereafter, whereas the contraction of responses in MCMV^M45Cterm^ infection was followed by a slight, but clearly noticeable inflation starting by 28 dpi (Fig 4B).

The long-term phenotype of the peptide-specific cells was CM in MCMV^WT^ infection, but EM upon MCMV^M45Cterm^ infection (Fig 4C–4E). Thus, C-terminal localization of the HGIRNASFI peptide within the M45 protein resulted not only in a stronger and inflating CD8 T-cell response, but also in a high percentage of peptide-specific cells with an EM phenotype at late time points after infection.

### C-terminal localization of the epitope improves processing and direct surface presentation

**Fig 5:**
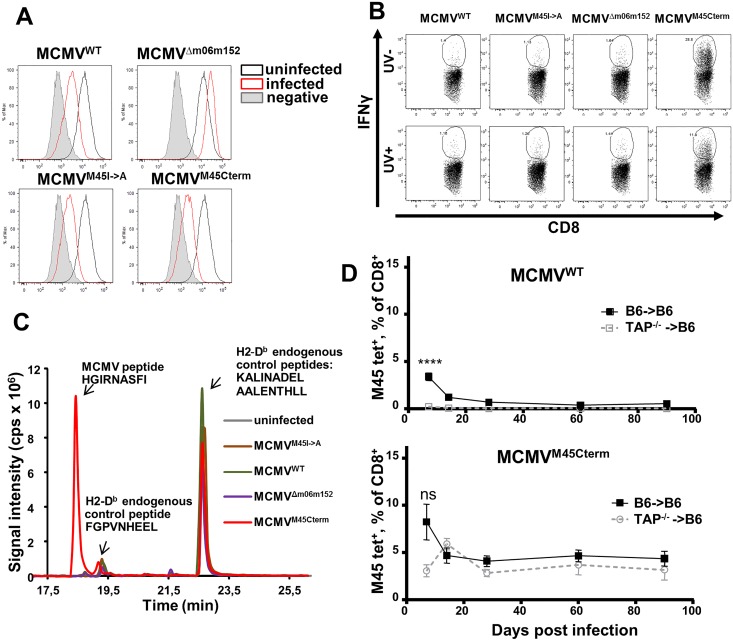
C-terminal localization of the HGIRNASFI peptide allows its presentation on the surface of infected cells. (**A**) Cell surface expression of MHC class I molecule (D^b^). LSECs (C57BL/6) were infected with the indicated viruses at MOI of 5 with centrifugal enhancement as described. D^b^ expression was measured by flow cytometry at 16h p.i‥ Fluorescence histograms for a representative experiment are shown. (**B**) LSECs were infected with the indicated viruses or incubated with a corresponding dose of UV inactivated virus (UV dose– 150J) at an MOI of 0.2 with centrifugal enhancement and co-cultured with HGIRNASFI-specific CTLs at an E:T ratio 3:1. Co-culture was performed for 15h, upon which the T cells were collected and stained for intracellular IFNγ. Where indicated, virus was inactivated by UV light. Two independent experiments were performed, with 4 or 5 wells per experimental condition. Representative dot plots are shown. (**C**) Relative intensity of signals measured by targeted nanoLC-MS^3^ from D^b^-immunoprecipitates of cells infected for 24 hours with indicated viruses at an MOI of 2 with centrifugal enhancement. Two high abundant endogenous control peptides (KALINADEL and AALENTHLL) and a low abundant (FGPVNHEEL) endogenous control peptide were present in all IP samples, indicating valid sample processing in all cases. The MCMV target peptide HGIRNASFI was detected only in the cells infected with the MCMV^M45Cterm^ recombinant. (**D**) Grouped means +/- SEM of blood CD8 T-cells stained by HGIRNASFI-D^b^ tetramers in bone-marrow chimeric mice, where C57BL/6 mice received TAP^-/-^ or C57BL/6 bone marrow at 3 months before infection with 10^6^ PFU/mouse of MCMV^WT^ (top panel) or MCMV^M45Cterm^ (bottom panel). The experiment was performed three times at 5 mice per group and pooled results are shown. Significance on day 7 p.i. was assessed by a Mann—Whitney *U* test. ****p < 0.0001, ns—not significant.

It was theoretically possible that virus mutagenesis and propagation resulted in unwanted mutations of immune evasion genes, improving peptide presentation and CD8 T-cell priming. To exclude this scenario, we compared MHC-I surface expression upon infection with MCMV^M45Cterm^, MCMV^M45I->A^, MCMV^WT^ and a mutant lacking the immune evasion genes *m06* and *m152* (MCMV^Δm06m152^). All of the MCMV recombinants, except MCMV^Δm06m152^, efficiently down-regulated MHC class I D^b^ molecules on infected liver sinusoidal endothelial cells (LSECs) (Fig 5A and S3A Fig). Thus, the inflationary EM response to the HGIRNASFI epitope encoded by MCMV^M45Cterm^ was not due to different surface levels of MHC-I. Since the HGIRNASFI epitope located at its native site is poorly processed and presented on infected fibroblasts [26], we considered that the improved response to MCMV^M45Cterm^ might be due to the availability of the peptide itself on MHC-I molecules. Hence, to measure the presentation of HGIRNASFI on LSECs, we analyzed the IFNγ response of a HGIRNASFI-specific CD8 T-cell line (CTL) upon co-culture with a recently published LSECs line [27]. LSECs infected with the control virus (MCMV^M45I->A^) or with MCMV^WT^ did not activate M45-specific CTL, whereas the MCMV^M45Cterm^ virus induced a robust activation (Fig 5B, **upper row**). Importantly, no CTL responses were observed upon infection with MCMV^Δm06m152^, demonstrating that the HGIRNASFI epitope expressed at its native site cannot activate CTL responses to infected LSECs even if MHC-I molecules are present at high levels on the cell surface. In theory, it was possible that an unknown MCMV gene that impairs the processing and presentation of the HGIRNASFI peptide in MCMV^WT^ infection was accidentally lost in the MCMV^M45Cterm^ mutant. Therefore, we UV-inactivated the viruses to abolish de novo gene expression and we co-cultured the cells exposed to UV-inactivated virus with M45-peptide-specific CTLs. Since M45 is a tegument protein, it is available in cells upon entry of UV-inactivated virus. As shown in Fig 5B **bottom row** and S3B Fig, inactivation of MCMV^WT^ and MCMV^Δm06m152^ did not result in recognition of the infected cells by the CTL, whereas MCMV^M45Cterm^ induced a measurable response, although somewhat weaker than upon infection with viable virus.

To conclusively show that HGIRNASFI localization within the M45 protein determines antigenic peptide availability on the D^b^ molecules of the infected cells, we performed a targeted nanoflow liquid chromatography mass spectrometry (nanoLC-MS^3^) analysis. Total D^b^ MHC-I molecules were immunoprecipitated (IP) from lysates of LSECs infected with MCMV^WT^, MCMV^M45I->A^, MCMV^Δm06m152^ or MCMV^M45Cterm^. Upon epitope elution from the pMHC-I complexes, IP samples were analyzed by targeted nanoLC-MS^3^ for the presence of the HGIRNASFI peptide.

As expected, the target peptide was not detected in the sample from MCMV^M45I->A^-infected cells. In line with previously published functional assays [26, 28], the peptide was also not detected in MCMV^WT^-infected cells, or in those infected with MCMV^Δm06m152^, although we detected traces of HGIRNASFI in one out of three repetitions of MCMV^WT^ and MCMV^Δm06m152^ infection. However, the target peptide was highly abundant in all 3 replicates of the MCMV^M45Cterm^-infected LSECs (Fig 5C) and its spectrum matched the one from the synthetic HGIRNASFI peptide (S3C Fig). Thus, the MS data confirmed that the C-terminal localization of the peptide facilitated its processing and its presentation in pMHC-I complexes.

Considering the very poor HGIRNASFI presentation on MCMV^WT^ infected cells, it is counterintuitive that the M45 epitope is immunodominant at 7 dpi [18]. However, this early immunodominance might be explained by peptide cross-presentation on professional antigen-presenting cells (APCs). This would imply that inflationary responses depend on epitope presentation on latently infected non-hematopoietic cells. To validate this idea, we generated chimeric mice with impaired MHC-I antigen presentation on professional APCs (but maintained on non-hematopoietic cells), by hematopoietic reconstitution of gamma-irradiated C57BL/6 recipients with TAP-deficient bone-marrow (BM) cells (TAP^-/-^→B6). Mice were infected with MCMV^M45Cterm^ or MCMV^WT^ and monitored for HGIRNASFI-specific responses. Additional controls included homochimeric mice where C57BL/6 mice were used both as BM donors and recipients (B6→B6). While B6→B6 mice showed kinetics that essentially matched the one observed in wild-type mice, the TAP^-/-^→B6 mice revealed a complete loss of HGIRNASFI response upon MCMV^WT^ infection (Fig 5D **top panel** and S3D Fig). In contrast, MCMV^M45Cterm^ induced strong CD8 T-cell responses against the peptide in TAP^-/-^→B6 mice (S3D Fig) that were undistinguishable from responses in B6→B6 mice at later times p.i. (Fig 5D **bottom panel**). Interestingly, the initial peak response, seen in WT mice or in B6→B6 controls was absent from the TAP^-/-^→B6 mice, which may indicate that this initial response is mainly driven by cross-presentation. Finally, one should note that approximately 90% of BM-derived cells in chimeric mice were donor-derived (S3E Fig). Therefore, the long-term CD8 T-cell response to the C-terminal epitope was maintained in absence of APC-dependent cross-presentation (Fig 5D), implying that it may depend on its direct presentation by virus-infected cells, although we cannot exclude the possibility that the initial priming was due to cross-presentation by the few remaining TAP-competent APCs derived from the recipient BM.

Taken together, our results showed that peptide localization within the M45 protein, rather than targeted activity of immune evasion genes, is the limiting factor for CTL recognition of the HGIRNASFI peptide in MCMV-infected LSECs.

### Role of peptide processing in the generation of inflationary responses

**Fig 6:**
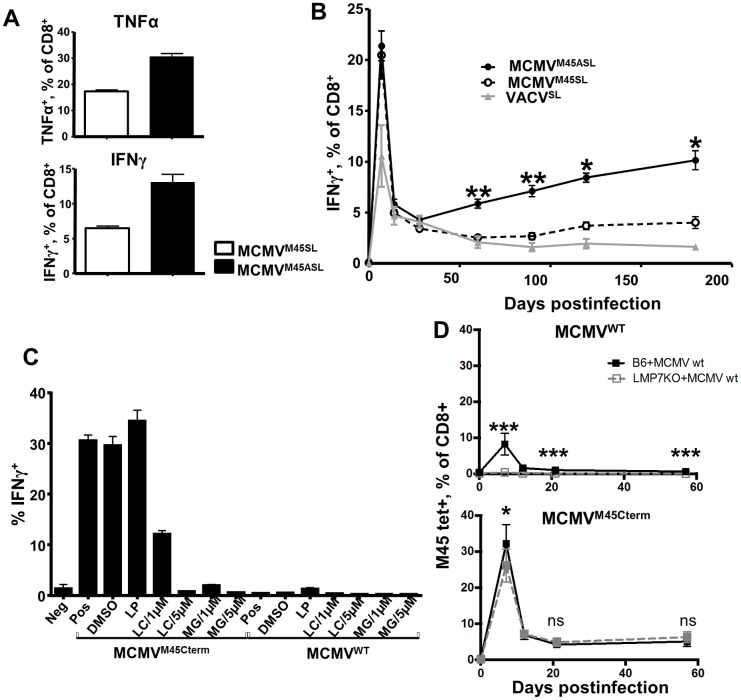
Constitutive proteasomal processing is critical for the induction of inflationary CD8 T-cell responses. (**A**) IC-21 cells were infected with indicated viruses at an MOI of 0.2 with centrifugal enhancement. Splenocytes obtained from gBT-I.1 mice were used as effector cells at an E:T ratio of 3:1. Splenocytes were not restimulated upon isolation from the mice and used untouched for the assay. Co-culture was performed overnight (15h). Columns represent the mean percentage of IFNγ^+^ or TNFα^+^ cells from triplicate experiments, and error bars show the SEM. (**B**) 129/Sv mice were infected intraperitoneally (i.p.) with 2x10^5^ PFU of indicated MCMV recombinants and 10^6^ PFU of VACV^SL^. Blood of mice was analysed for the fraction of CD8 T cells responding to *in vitro* re-stimulation with the SSIEFARL peptide for 6 hours, followed by intracellular staining for IFNγ. Grouped means +/- SEM of cells responding to the SSIEFARL peptide at 7, 14, 28, 60, 90, 120, 180 dpi are shown. The experiment was performed two times independently, at 5 mice per group in each experiment, and grouped averages from two experiments are shown. Difference in responses between groups infected with either MCMV^M45SL^ or MCMV^M45ASL^ was identified. Significance was assessed by Kruskal-Wallis test followed by Dunns post-analysis for MCMV^M45SL^ and MCMV^M45ASL^ infected mice (*p<0.05, **p<0.01). (**C**) Treatment with proteasomal inhibitors (but not protease inhibitors) impairs target cell recognition by HGIRNASFI-specific CTL. Target cells (LSECs) were pretreated for 5h with indicated inhibitors, washed twice with PBS and infected with MCMV^WT^ or MCMV^M45Cterm^ at an MOI 0.2 with centrifugal enhancement. Co-culture with HGIRNASFI-specific CTLs was performed at an E:T ratio 3:1 for 15h, upon which the T cells were collected and stained for intracellular IFNγ. The y-axis shows percentages of CTL responding by IFNγ to co-culture with target cells (mean +/- SEM from three experiments is shown). Labels below the x-axis show the deployed inhibitor and its concentration in μM; LC—lactacystin, MG—MG132, LP—leupeptin; Pos–positive control, target cells infected with indicated viruses without pretreatment with inhibitors; DMSO—infection in presence of the diluent for inhibitors Neg–negative control, untreated cells. (**D**) LMP7^-/-^ and C57BL/6 adult mice were infected i.v. with 10^5^ PFU of MCMV^WT^ (top panel) or MCMV^M45Cterm^ (bottom panel). The percentage of blood CD8 T cells stained by HGIRNASFI-D^b^ tetramers was measured at the indicated time points. The data show the mean values +/- SD from of pooled results from 2 independent experiments (in total 8 mice per each group) Significance on indicated time points was assessed by a Mann—Whitney *U* test. *p < 0.05. ***p < 0.0001, ns—not significant.

Since the MHC-I availability of the same peptide expressed by the same viral gene differed greatly based on its localization within the protein, we considered that this difference may be due to improved peptide processing prior to loading on MHC-I molecules. Antigenic peptide processing and subsequent surface presentation of peptide MHC-I complexes can be enhanced by altering amino acid residues flanking an epitope [29]. Thus, we generated a novel MCMV recombinant called MCMV^M45ASL^, carrying the SSIEFARL peptide at the C-terminus of the M45 protein, but preceded by 2 alanines (S4A Fig). Thus, these two alanines were the only difference between MCMV^M45ASL^ and MCMV^M45SL^ [8]. The *in vitro* and *in vivo* growth of MCMV^M45ASL^ was comparable to MCMV^WT^ (S4B Fig). To test the effect of these flanking residues on epitope recognition by CD8 T cells, we co-cultured CD8 T cells from transgenic gBT-I mice expressing a T-cell receptor specific to the K^b^-SSIEFARL complex [30], with IC-21 macrophages infected with MCMV^M45SL^ or MCMV^M45ASL^, and assessed them for TNFα and IFNγ production. Infection with the recombinant MCMV containing the alanine spacer induced a stronger T-cell response than the one without it (Fig 6A). The same result was observed upon co-culture with *in vitro* infected LSECs (S4C Fig). This implied that peptide processing is a rate limiting step in activating CD8 T cells by our mutants. Therefore, we tested if the same would apply *in vivo* and affect the size of inflationary responses to SSIEFARL. We infected mice with MCMV^M45SL^, MCMV^M45ASL^ or, as a non-inflationary control, with rVACV^SL^. At 7 dpi, the percentage of SSIEFARL-responding cells was identical in mice infected with either MCMV recombinant (Fig 6B). Likewise, both mutants induced SSIEFARL responses with inflationary phenotype (S4D Fig). On the other hand, the peptide-specific CD8 T cells showed an inflationary trend only in mice infected with the MCMV^M45ASL^ recombinant (Fig 6B), and differences in peptide-specific responses were statistically significant at all times after 60 dpi. Hence, improved peptide processing resulted in stronger MI.

To test if C-terminal expression improved the processing of peptides by the proteasome, MCMV^M45Cterm^ or MCMV^WT^-infected LSECs were co-cultured with CTLs in the presence of two proteasome inhibitors—MG-132 or Lactacystin. Both inhibitors impaired CTL activation upon co-culture with cells infected with the MCMV^M45Cterm^ recombinant in a dose dependent manner (Fig 6C). On the other hand, CTL activation by cells that were loaded exogenously with the peptide remained unimpaired (S4E Fig) and MCMV^WT^ infection did not activate CTLs in presence or absence of the inhibitors (Fig 6C). Finally, treatment with a protease inhibitor (leupeptin) did not influence CTL recognition of the MCMV^M45Cterm^-infected target cells (Fig 6C), confirming that the C-terminally expressed epitope was proteasomally processed for CTL recognition. We infected mice lacking the immunoproteasome component LMP7 with MCMV^WT^ or MCMV^M45Cterm^, and compared the kinetics of their HGIRNASFI responses to the parental C57BL/6 strain. The response was absent in LMP7^-/-^ mice upon MCMV^WT^ infection (Fig 6D **top panel**), but fully maintained upon MCMV^M45Cterm^ infection. (Fig 6D **bottom panel**). Interestingly, the acute response at 7 dpi with MCMV^WT^ was completely abrogated (*p<0*.*001*) in LMP7^-/-^ mice (Fig 6D **top panel**), but only slightly impaired (*p<0*.*05*) upon MCMV^M45Cterm^ infection (see Fig 6D **bottom panel**). This argued that even the acute CD8 T-cell response was largely immunoproteasome independent when the epitope was relocated to the C-terminal position. Taken together, our data strongly argued that the efficacy of peptide processing defines the rate of CD8 T-cell inflation for a given peptide in MCMV infection.

## Discussion

We reported here several findings that were counterintuitive in light of the existing literature. Previous publications showed that immune sensing of an antigenic epitope may impair the transcription of viral genes expressed later in the process of viral reactivation [31], and that antigen-specific inflationary responses compete with each other [8, 32]. Taken together, the evidence was unified in the Immune Sensing Hypothesis [20], where intermittent MCMV transcription during viral latency results in antigen expression, T-cell sensing and suppression of genes expressed later during reactivation, thus defining the immunodominance hierarchy. On the other hand, the Immune Sensing Hypothesis did not explain the inflationary responses against epitopes expressed by the early genes *M38* or *m139* [18]. We showed here that the conventional responses against the natural M45 epitope are not due to its silenced transcription in latency. In fact, we showed that the very same viral gene can simultaneously induce inflationary and conventional responses to two epitopes expressed within its protein product, thus demonstrating that promoter activity cannot alone predict which epitopes are inflationary. We observed that sequences flanking an antigenic peptide and its position within a protein may critically define the type of responses and that the efficacy of peptide processing by the constitutive proteasome is a rate-limiting factor for MI. Our results are not necessarily in conflict with the immune sensing hypothesis, but rather complement it with a secondary mechanism defining epitope dominance. Namely, while we show that immunodominance and MI depend on processing efficacy, the epitopes cannot be antigenic unless expressed, and hence the pattern of gene expression during viral latency is necessarily an important contributing mechanism [8, 20].

Previous publications showed that CTL priming upon HSV-1 infection requires cross-presentation [33], and that the same might be true for the MCMV responses [34, 35], but we showed that this holds true only for conventional CTL responses against a non-inflationary epitope. Transferring the conventional HGIRNASFI epitope to the C-terminus of the M45 protein allowed its direct presentation on virus-infected LSECs, and induced inflationary responses. Impaired cross-presentation (Fig 5D) abrogated the conventional HGIRNASFI response, but not the inflationary one. Previous data showed that non-hematopoietic cells, such as LSECs, are sites of MCMV latency [36, 37] and that peptide presentation on non-hematopoietic cells is required for inflationary, but not for conventional immune responses [38, 39]. However, our work differs in experimental design from published evidence in two important aspects, and thus allows us to posit novel conclusions. Firstly, we generated recombinant viruses that allowed us to compare the response to the same epitope expressed in two different locations of the same gene, while published evidence was based on cross-comparison of two distinct MCMV epitopes, expressed by different viral genes. Therefore, previous evidence could have been explained by peptide-intrinsic properties, or by cell-type specific differences in promoter activity, whereas our data excluded these scenarios and allowed us to focus on processing as the determinant of memory inflation. Secondly, we performed bone-marrow reconstitution of C57BL/6 mice with TAP deficient bone-marrow, while previous work was based on MHC-deficient recipient mice of wild-type bone marrow. Therefore, prior evidence showed that direct presentation is required for memory inflation, whereas our work might indicate that it is sufficient for this phenomenon. Namely, while the response to the HGIRNASFI epitope in wild-type MCMV infection required cross-presentation, because it was completely abrogated in mice transferred with TAP^-/-^ bone-marrow, responses were undiminished when the epitope was expressed from the C-terminus, arguing that cross-presentation on APC might be dispensable for the induction of inflationary responses. If that was true, our result would point to direct priming by antigen presented on non-hematopoietic cells, which would defy a key dogma in immunology. It is important to note, however, that the irradiation and bone-marrow transfer procedure resulted in chimerism, where approximately 10% of PBMCs remained TAP-competent (S3E Fig). While this was not enough for the priming of conventional CD8 T cell responses in the setup of infection with MCMV^WT^, our data cannot exclude the possibility that the few remaining TAP-competent cells were sufficient to provide cross-priming and jump-start the inflationary immune response. In conclusion, while our data are intriguing, more detailed analyses are necessary to ascertain if inflationary responses may occur in the absence of an initial bout of cross-priming.

Previous evidence showed that the immunoproteasome is not strictly required for processing of inflationary epitopes [40]. Similarly, a recent publication showed that the non-inflationary HGIRNASFI epitope may induce inflationary responses when expressed as a minigene within an adenovirus-based vaccine vector [41]. However, these experiments could still be explained within the Immune Sensing Hypothesis, where memory inflation is predicated by the context of gene-expression of the antigenic epitope. By testing the same epitope in the context of the same protein, we excluded confounding factors and showed directly that peptide processing by the constitutive proteasome and direct antigen presentation on non-professional APC are the rate-limiting events for MI.

Predictive algorithms to identify epitope insertion sites with optimized antigenic processing [42], have been recently applied to score peptide processing of natural and modified epitopes expressed by MCMV [43]. However, published data argue that the proteasome needs to cleave peptides very precisely behind the anchoring amino acid at the C-terminus of an epitope (reviewed in [44]), whereas additional amino acids present on the N-terminal end of the epitope precursor peptide may be trimmed by aminopeptidases upon TAP-mediated transport into the ER [45]. Therefore, designing vectors that already carry the antigenic peptide at the C-terminus of a protein abrogates the need for proteasomal cleavage on the sensitive C-terminus of the peptide and thereby substantially improves the inflationary CD8 T-cell response. This insight likely explains why the surface presentation of the HGIRNASFI peptide, that T cells do not recognized *in vitro* (Fig 5B) or *in vivo* [24] at its native site, could be substantially optimized by this tweak in vector design. Accordingly, the E7 antigen, just like the prostate specific antigen [7], induced weak CD8 T-cell responses and poor protection when expressed within full length proteins in recombinant MCMVs, but a single E7 peptide fused to the C-terminus of the ie2 MCMV protein induced not only robust CD8 T-cell responses, but also absolute anti-tumor immunity (Fig 1). Similarly, SSIEFARL epitopes expressed on the C-terminus of two MCMV proteins provided protection against viral challenge (Fig 2A). In conclusion, our data showed that simple shifting of an epitope sequence to the C-terminus of a protein optimizes peptide processing and inflationary CD8 T-cell responses and circumvents the need for predictive algorithm scoring of the epitope insertion site.

Although our results unequivocally demonstrate that response magnitude and MI depend on the peptide context within a viral protein, one should take into account that additional parameters are likely to contribute to memory inflation and epitope immunodominance. These may include peptide intrinsic properties, such as its avidity of binding to MHC-I molecules and the avidity of TCR binding to pMHC complexes [46], but also extrinsic properties, such as the promoter strength and epitope competition [8, 31, 32], or the expression of additional viral genes, as has been recently described in the rhesus CMV model of infection [47]. Therefore, the mechanisms driving the exceedingly strong CMV responses remain a field of active research. Considering the huge potential that CMV-based vectors may have for the control of numerous lethal pathogens [9, 12, 13], understanding the mechanisms that optimize antigen presentation and the induction of T-cell responses remains of paramount scientific and clinical relevance. By providing the first direct evidence for a causal link between antigen processing efficacy, epitope presentation, memory inflation and immune protection, our study makes a fundamental contribution towards this goal.

## Materials and Methods

### Ethic statement

Mice were housed and handled in accordance with good animal practice. All animal experiments involving HPV immunization and challenge were performed at OHSU according to federal (U.S. Animal Welfare Act) and institutional guidelines, following the Institutional animal care and usage committee (IACUC) requirements, under the protocol (IACUC Study #IS00003413). Experiments involving LMP7^-/-^ mice were performed according to U.K. Animal Welfare Act of 2006 and Home Office regulations (project license no. PPL 30/3293) after review and approval by the local Ethical Review Board at the University of Oxford. All other animal experiments were performed at HZI in compliance with the German animal protection law (TierSchG BGBI S. 1105; 25.05.1998) and were approved by the responsible state office (Lower Saxony State Office of Consumer Protection and Food Safety) under permit number 33.9-42502-04-11/0426. The mice were housed and handled in accordance with good animal practice as defined by FELASA and the national animal welfare body GV-SOLAS.

### Animal strains

129S2/SvPas Crl (129/Sv) mice were purchased from Charles River (Sulzfeld, Germany). C57BL/6 mice were purchased from Janvier (Le Genest St Isle, France) or Jackson Laboratory (Sacramento, CA, USA). Mice used for generation of bone marrow chimeras (B6.129S2-*Tap1*^*tm1Arp*^/J, C57BL/6J, B6.SJL-*Ptprc*^*a*^ *Pepc*^*b*^/BoyJ) were purchased from The Jackson Laboratory (Sacramento, CA USA). gBT-I.1 mice [30] were a gift from G. Behrens. LMP 7^-/-^ mice on a C57BL/6 background [48] were bred and housed at the University of Oxford Biomedical Sciences Specified Pathogen Free (SPF) Facility. Age-matched female C57BL/6 mice (Harlan, Bicester UK) were used as controls.

### Cells

M2-10B4 (CRL-1972; ATCC), and NIH 3T3 fibroblasts (CRL-1658; ATCC) were maintained in DMEM supplemented with 10% fetal calf serum, 1% Glutamine and 1% Penicillin/Streptomycin. IC-21 macrophages (TIB-186; ATCC) were maintained in RPMI 1640 medium supplemented with 10% fetal calf serum, 1% Glutamine and 1% Penicillin/Streptomycin. TC-1 tumor cells were grown as described [22]. LSECs from C57BL/6 mice were generated and maintained as described [27]. C57BL/6 murine embryonic fibroblasts (MEFs) were prepared and maintained as described previously [49].

### Peptides, primers

The peptides M45 (H-2D^b^-restricted, ^985^HGIRNASFI^993^) and the HSV-1 glycoprotein B-derived epitope (H-2K^b^-restricted, ^498^SSIEFARL^505^) [50] were synthesized and HPLC purified (65–95% purity) at the HZI peptide-synthesis platform. Primers used in the study are listed in the S1 Table.

### Viruses and viral mutagenesis

MCMV^M45ASL^, MCMV^M45Cterm^, MCMV^M45I->A^ and MCMV^ie2E7^ recombinants were generated by En passant mutagenesis as described by Tischer and colleagues [51], with modifications described by us [52]. In brief, linear PCR products were generated using the plasmid pGP704 I-SceIKan [52] as template and primers containing MCMV-homologue sequences and constructs to be introduced in the MCMV genes on their 3’ ends. Each linear construct was generated by 2 rounds of PCR amplification [52], and contained sequences of the antigenic peptides inserted in the MCMV genome and sites of homologies allowing targeting to specific sites in the virus genome. All primers used in the study are listed in S1 Table. Upon cloning on chloramphenicol LB-agar plates insertion was confirmed by colony PCR and sequencing of the insertion site.

To generate MCMV^E6+E7^, the fragments containing the entire E6 and E7 genes of HPV16 were cloned into pOriR6K-ie-zeo [53]. The resulting plasmid pO6-ie-E6+E7 was inserted into pSM3fr-Δ1-16-FRT [21] via *Flp*-mediated recombination [53] resulting in pSM3fr-Δ1-16-FRT- ie-E6+E7. The same E6 and E7 gene sequence was fused on the 3’-end of the *ie2* gene in a pSM3fr MCMV BAC [54] by homologous recombination to generate pSM3fr-ie2E6+7. All viruses used in this study are listed in S2 Table.

Reconstitution of MCMV from recombinant BACs was done by transfection of MEFs as described previously [8]. BAC-derived wild-type [55] and all recombinant MCMVs used in the study were propagated on M2-10B4 cells and virus stocks were purified on sucrose gradient as described previously [37]. Virus stocks were titrated on MEFs as described before [8]. MCMV from organ homogenate or tissue culture supernatants were titrated on MEFs as described previously [56].

Recombinant VACV expressing an immunodominant peptide from HSV-1 (VACV^SL^) was obtained from Dr. J. Nikolich-Zugich, University of Arizona, and grown on Vero cells [57, 58].

### In vitro infections

*In vitro* growth fitness of the recombinant viruses was determined on NIH 3T3 cells as described before [8]. For infection with centrifugal enhancement, plates were centrifuged at 2000rpm for 30 min, incubated at 37°C, 5% CO_2_ for another 30 min, upon which the supernatants were replaced with fresh medium. This procedure increases virus infection by a factor of 20 and thus a nominal MOI of 0.2 with centrifugal enhancement equals an MOI of 4 without it.

### In vivo infection

Mice (6–12 weeks old) were intraperitoneally infected and housed in SPF conditions throughout the experiment. Infected mice showing very weak immune priming (2 standard deviations below average) at 7 dpi were regarded as outliers due to suboptimal infection and were excluded from the study.

### Immune protection assays

129Sv mice were i.p. infected with 2x10^5^ PFU of MCMV^WT^ or MCMV^M45SL^. Eight months later, mice were i.p. challenged with 10^6^ PFU of recombinant VACV^SL^. Ovaries were harvested at 7 days post challenge and infectious VACV titers established by plaque assay on Vero cells. For tumor challenge experiments animals were i.p. primed and boosted at 4 weeks interval with recombinant MCMV as indicated. Prior to challenge with 2.5x10^4^ exponentially growing TC-1 tumour cells [22] mice were shaved on the hind left flank and s.c. injected. Mice were followed for tumor growth and tumor volume calculated by length x height x 0.5.

### Generation of bone marrow chimeras (BMC)

Bone marrow cells were isolated from tibias and femurs of B6.129S2-*Tap1*^*tm1Arp*^/J or C57BL/6J mice (CD45.2 expressing), depleted of T cells with anti-PE MicroBeads (Milteniy Biotec) and CD90.2-PE (BioLegend) antibody according to the manufacturer protocol. Recipient mice (B6.SJL-*Ptprc*^*a*^ *Pepc*^*b*^/BoyJ) were gamma-irradiated with a lethal dose of 9.5–10 Gγ, and 6–8 hours upon irradiation received 3–5×10^6^ bone marrow cells. Within the first two weeks upon reconstitution, mice were prophylactically treated with enrofloxacin. Experiments on BMC mice were performed 12 weeks after reconstitution.

### M45 (D^b^) CTL generation

Splenocytes from C57BL/6 mice (latently (>3 months) infected with MCMV^WT^), were isolated for CTL generation as described before [49] with minor modifications. IL-2 was added at a concentration 200U/ml, the peptide at a concentration 10^-10^M. CTL were used after the 2^nd^ round of re-stimulation.

### Co-culture experiments

Target cells were seeded in 96-well plates. Infection was performed at an MOI 0.2 with centrifugal enhancement (~MOI 4 in standard conditions) as described above. After 1h infection, infectious supernatants were removed and effector cells (either *in vitro* generated M45(D^b^) or *ex vivo* harvested gBT-I.1 CD8 T-cells) were added at an E:T ratio of 3:1. The cells were incubated at 37°C for 1h, upon which brefeldin A (Cell Signaling Technology) was added at a concentration 10μg/ml and cells were incubated for 14h. For proteasomal inhibition experiments, target cells were pretreated for 5h with indicated inhibitors.

### Liquid chromatography-mass spectrometry analysis (LC-MS)

LSECs were infected with indicated viruses at an MOI 2 with centrifugal enhancement. Cells were harvested 24 hpi and cell lysates were prepared with complete RIPA buffer with 2x protease inhibitor cocktail mix (Roche). The lysates were sonicated with Bioruptor Plus at low intensity and MHC-I molecules were immunoprecipitated with GammaBind Plus Sepharose Beads (GE Healthcare) coupled with anti-mouse H-2D(b) (Clone 28-14-8, BD Pharmingen). Immunoprecipitates were stored at -70°C until MS analysis. Peptides were eluted from the immunoprecipitated pMHC complexes with 0.2% trifluoroacetic acid (TFA) in water, subjected to ultrafiltration with a cut-off of 10 kDa (Vivacon 500 filters, Sartorius Stedim Biotech), desalted with OMIX C18 10–100 μL pipette tips (Agilent Technologies) and vacuum dried. Samples were re-suspended in 3% acetonitrile (ACN), 0.1% formic acid (FA) and 0.01% TFA in water prior to LC-MS analysis.

Nano ultra-performance liquid chromatography mass spectrometry (nano-UPLC-MS) analysis was performed using a NanoAcquity UPLC system (Waters Corp.) coupled to a QTRAP6500 (AB SCIEX) mass spectrometer equipped with a nano-ESI (electron spray ionization) source. Samples were separated on a nanoAcquity UPLC BEH C18 analytical column (0.075 x 250mm) (Waters). The LC separation started with 97% eluent A (0,1% FA and 0,01% TFA in water) to 10% eluent B (0,1% FA and 0,01% TFA in ACN) by 1 min with a linear gradient and then to 40% eluent B by 50 min with a linear gradient. The flow rate was set to 300nL/min. The mass spectrometer was operated in a low mass hardware profile operating in positive mode. The nano-ESI voltage was set at 2700 V, curtain gas at 30 L/min, ion source gas at 15 L/min, collision gas (CAD) high and interface heater temperature at 150°C. The resolution of the first (Q1) and third quadrupole (Q3) was set at unit resolution.

Synthetic reference peptides for target and control epitopes were provided by the in-house DKFZ core facility with an analytical purity of >95%. Known cytoskeletal and housekeeping-protein derived H2-D^b^-restricted epitopes were used as positive controls [59, 60].

A minimum of four fragments with the best signal-to-noise ratio were assigned per peptide during direct injection of synthetic peptides. Critical MS parameters (e.g., declustering potential, collision energy) were manually optimized to achieve the best sensitivity for the following peptides (precursor ion m/z: fragment ion m/z): HGIRNASFI (507.78: y_8_ 877.49; b_8_^2+^ 442.23; a_8_^2+^ 428.23; b_7_-H_2_O 718.37; b_7_ 736.38); FGPVNHEEL (521.25: y_7_^2+^ 419.21; b_8_^2+^ 455.71; y_5_ 641.29; y_8_ 894.43); KALINADEL (493.78: b_8_ 855.46; MH-H_2_O^2+^ 484.77; b_7_ 726.41; b_6_ 611.39); AALENTHLL (491.27: b_5_-NH_3_ 482.22; y_7_^2+^ 420.23; y_7_ 839.46; y_6_ 726.38).

The MS results of synthetic peptides were manually compared to the MS results acquired in the IP sample using the Analyst 1.6.2 (AB SCIEX) software. Identity of the targeted peptides was confirmed by their retention times, chromatographic profiles and MS^3^ spectra of each fragment. Targeted nanoLC-MS^3^ was performed to ensure sufficient sensitivity and specificity of MS results. Retention times (Fig 5C) and MS^3^ spectra (S3C Fig) of the IP samples were compared to the results of the synthetic HGIRNASFI reference peptide.

### Cell surface, intracellular staining and flow cytometry

Blood sample collection, processing and subsequent stimulation of blood lymphocytes with indicated peptides and intracellular cytokine staining was performed as described before [8]. The antibody panels used for the samples stimulated with the SSIEFARL peptide or stained with SSIEFARL-Kb tetramers shown previously [8] were expanded with anti-CD62L-eFluor605NC (MEL-14, eBioscience) for tetramer staining or with anti-KLRG1-Biotin (Clone 2F1; Biolegend) and anti-CD127-PE (Clone A7R34; Biolegend) for peptide re-stimulation. For intracellular cytokine staining of the samples stimulated with the HGIRNASFI peptide we used the following antibody panel: anti-CD4-Pacific Blue (Clone GK1.5; Biolegend); anti-CD8a-PerCP/Cy5.5 (Clone 53–6.7; Biolegend); anti-CD44-Alexa Fluor 700 (Clone IM7; Biolegend); anti-CD11a-PE-Cy7 (Clone 2D7; BD Bioscience); anti-CD3-APC-eFluor 780 (Clone 17A2, eBioscience), anti-CD127-PE (Clone A7R34; Biolegend), anti-KLRG1-Biotin (Clone 2F1; Biolegend), Streptavidin-Briliant Violet-570 (Biolegend). For intracellular staining we used anti-IFNγ. Following antibodies were used for characterization of the immune response in bone marrow chimeras: anti-CD4-Pacific Blue (Clone GK1.5; Biolegend); anti-CD8a-PerCP/Cy5.5 (Clone 53–6.7; Biolegend); anti-CD44-Alexa Fluor 700 (Clone IM7; Biolegend); anti-CD11a-PE-Cy7 (Clone 2D7; BD Bioscience); anti-CD3-APC-eFluor 780 (Clone 17A2, eBioscience); HGIRNASFI-Db tetramers-APC. For responses to the HPV-E7 epitope cells were by stained with anti-CD8a PerCP-Cy5.5 (clone 53–6.7; BD Bioscience), anti-CD4-FITC (clone RM4-5; Biolegend), H-2D(b) E7 (RAHYNIVTF) PE Dextramer (Immudex). In experiments on LMP7^-/-^ mice, we used anti-mouse CD8, CD44, KLRG-1, CD27, CD127 and CD62L (all eBioscience) and the Live/dead fixable near-infrared dead cell stain kit (Life-Technologies, Paisley, UK). The effector cells from co-culture experiments were transferred in fresh 96-wells and stained with the following antibodies: anti-CD4-Pacific Blue (Clone GK1.5; Biolegend); anti-CD8a-PerCP/Cy5.5 (Clone 53–6.7; Biolegend); anti-IFNγ-APC (Clone XMG1.2; Biolegend) and anti-TNFα-FITC (Clone MP6-XT22, BioLegend). For quantification of MHC class I expression on LSECs or MEFs, cells were trypsinized, washed in 1xPBS and stained for 30 min with anti-MHC-I (H-2Db)-PE (Clone 28-14-8, Biolegend). Cells were acquired in BD LSR-II or BD LSRFortessa cytometers (BD Bioscience). Cytometric results were analyzed with FlowJo software (version 9.5.3).

### Statistics

Statistical analysis was performed using GraphPad Prism program (version 5.04). Kruskal-Wallis analysis followed by Dunns post-analysis was used to compare multiple samples at single time points. Comparisons between two groups were performed using the Mann—Whitney *U* test (two-tailed).

## Supporting Information

S1 Fig (**A**) CL57BL/6 mice were vaccinated with 10^6^ PFU/mouse of MCMV^E6+E7^. 10 weeks later splenocytes were harvested and incubated for 36 hours with the indicated peptides and responses assayed by IFNγ ELISPOT. Neo_49-59_ is an irrelevant D^b^-restricted peptide (SSPVNSLRNVV) used as a negative control. IE3^416-423^ is an endogenous inflationary epitope from the *IE3* MCMV gene, used as a positive control. (**B**) 129/Sv mice were infected intraperitoneally (i.p.) with 2x10^5^ PFU of MCMV^M45SL^. Blood leukocytes were collected at 7, 14, 28, 60, 90, 120, 180 dpi and surface stained with the SSIEFARL tetramer and following antibodies: CD3, CD4, CD8, CD11a, CD44, CD62L and analyzed by flow cytometry. Graphs represent epitope-specific cells with the EM (CD62L^-^CD44^+^) or the CM (CD62L^+^CD44^+^) phenotype. The experiment was performed once, at 5 mice per group, and grouped averages +/- SEM are shown.
(PPTX)

S2 Fig The MCMV genome area between kilobases 58–59 corresponds to the MCMV gene *M45* (enlarged). (**A**) In order to prevent MHC class I presentation of the endogenous HGIRNASFI epitope, its anchoring amino acid (isoleucine) was swapped with the irrelevant amino acid (alanine), which cannot efficiently interact with the peptide-binding cleft of the MHC class I molecule. This resulted in generation of the MCMV^M45I->A^ mutant. (**B**) A construct AAHGIRNASFI was inserted by means of traceless BAC mutagenesis at the very end of the *M45* gene of MCMV^M45I->A^ recombinant (the DNA nucleotide sequence (black letters) as well as the corresponding amino acid sequence (grey letters) are shown). (**C**) *In vitro* growth kinetic of MCMV^M45I->A^ and MCMV^M45Cterm^ on NIH3T3 cells. A monolayer of NIH3T3 cells was infected in three independent experiments with indicated viruses at an MOI of 0.1. Medians at indicated time points post infection are shown, vertical bars show standard deviations. (**D**) Swapping of amino acids in the immunodominant M45D^b^-restricted peptide and insertion of the peptide in the C-terminus of the M45 protein does not influence viral growth *in vivo*. 129/Sv mice were i.p. infected with 2x10^5^ PFU of indicated virus. Spleen, liver and lung homogenates were assayed for infectious MCMV titer at day 5 p.i‥ Salivary gland homogenates were assayed at 21 day p.i‥ Each symbol represents one mouse, horizontal lines indicate medians.
(PPTX)

S3 Fig (**A**) D^b^ expression represented as geometric mean fluorescent intensity (GMFI) of the corresponding Ab signal for the experiment shown in Fig 5A. The experiment was performed twice in triplicates and histograms represent the geometric mean values of all data. Error bars show the SEM. (**B**) Group averages of IFNγ responding cells shown as representative dot blots in Fig 5B. Error bars show the SEM, “+” or “–” indicate respectively whether virus was UV inactivated prior infection or not. (**C**) MS identity confirmation of target peptide HGIRNASFI. The MS^3^ spectrum for fragment a_8_^2+^ (507.78/428.23) of the synthetic reference peptide HGIRNASFI (lower panel) matches the MS^3^ spectrum of the peptide identified in the IP sample (upper panel) of MCMV^M45Cterm^ infected cells (eluting at app. 18.5 min, see Fig 5C). MS^3^ spectra were detected for all four fragments, confirming the presence of the peptide HGIRNASFI in the IP sample. Only one fragment MS^3^ spectrum is shown for clarity reasons. (D) Detailed representation of the M45-specific CD8 T-cell response on day 7 p.i. for the experiment shown in Fig 5D. Each symbol represents a mouse, horizontal lines denote group means. Significance was assessed by Mann-Whitney test. ****—p<0.0001, ns—not significant. (E) Bone marrow chimera mice were generated as described in material and methods section. On day 70 upon bone marrow transfer mice were bled via retro orbital route and percentage of CD45.2+ CD8 T cells (donor cells) in the blood samples was identified by flow cytometry as a marker of chimerism.
(PPTX)

S4 Fig (**A**) The MCMV genome area between kilobases 58–59 corresponds to the MCMV gene *M45* (enlarged). A construct AASSIEFARL or SSIEFARL was inserted by means of traceless BAC mutagenesis at the very end of the *M45* gene of MCMV^WT^ (the DNA nucleotide sequence (black letters) as well as the corresponding amino acid sequence (grey letters) are shown). (**B**) Growth fitness of MCMV^M45ASL^ mutant compared to MCMV^WT^. Left graph: C57BL/6 mice were i.p. infected with 10^6^ PFU of indicated virus. Spleen homogenates were assayed for infectious MCMV titer at day 5 p.i‥ Each symbol represents one mouse; horizontal lines indicate medians. Right graph: *in vitro* growth kinetic of MCMV^M45ASL^ on NIH3T3 cells. A monolayer of NIH3T3 cells was infected in three independent experiments with indicated viruses at an MOI of 0.1. Medians at indicated time points post infection are shown, vertical bars show standard deviations. (**C**) LSECs were infected with indicated viruses at an MOI of 0.2 with centrifugal enhancement. Splenocytes obtained from gBT-I.1 mice were used as effector cells at an E:T ratio of 3:1. Splenocytes were not restimulated upon isolation from the mice and used untouched for the assay. Co-culture was performed overnight (15h). Columns represent the mean percentage of IFNγ^+^ cells from 3 independent experiments, and error bars show the SEM. (**D**) SSIEFARL-specific CD8 T cells (IFNγ^+^ secreting) from experiment shown in Fig 6B were analysed for the surface expression of CD127 and KLRG1. The staining was used to define the CM (CD127^+^KLRG1^-^) and the EM (CD127^-^KLRG1^+^) subsets. Grouped means +/- SEM of the percentage of EM (upper graph) or CM (lower graph) cells in the SSIEFARL-responding subset at indicated time points p.i‥ Significance on day 180 p.i. was assessed by Kruskal-Wallis test followed by Dunns post-analysis for MCMV^M45SL^ and MCMV^M45ASL^ infected mice (ns—not significant). (**E**) Treatment with proteasomal inhibitors does not impair CTL recognition of HGIRNASFI peptide-pulsed target cells. Target cells (LSECs) were pretreated for 5h with indicated inhibitors, washed twice with PBS and pulsed for 1h with HGIRNASFI peptide at concentration 1μg/ml. The y-axis shows percentages of CTL responding by IFNγ to co-culture with target cells (mean +/- SEM from three experiments is shown). Labels below the x-axis show the concentrations of deployed inhibitor in μM; “+”–positive control, target cells pulsed with the peptide without pretreatment with inhibitors; “-”–negative control, untreated cells.
(PPTX)

S1 Table List of all primers used in the study. (DOCX)

S2 Table List of all recombinant viruses used in the study. (DOCX)
