# Supplementary material for: Peptide Processing Is Critical for T-Cell Memory Inflation and May Be Optimized to Improve Immune Protection by CMV-Based Vaccine Vectors
Source: PLoS Pathog. 2016 Dec 15;12(12):e1006072. doi: 10.1371/journal.ppat.1006072 (PMC5158087; doi:10.1371/journal.ppat.1006072)
Supplement: S1 Fig — (A) CL57BL/6 mice were vaccinated with 106 PFU/mouse of MCMVE6+E7. 10 weeks later splenocytes were harvested and incubated for 36 hours with the indicated peptides and responses assayed by IFNγ ELISPOT. Neo49-59 is an irrelevant Db-restricted peptide (SSPVNSLRNVV) used as a negative control. IE3416-423 is an endogenous inflationary epitope from the IE3 MCMV gene, used as a positive control. (B) 129/Sv mice were infected intraperitoneally (i.p.) with 2x10^5^ PFU of MCMVM45SL. Blood leukocytes were collected at 7, 14, 28, 60, 90, 120, 180 dpi and surface stained with the SSIEFARL tetramer and following antibodies: CD3, CD4, CD8, CD11a, CD44, CD62L and analyzed by flow cytometry. Graphs represent epitope-specific cells with the EM (CD62L-CD44+) or the CM (CD62L+CD44+) phenotype. The experiment was performed once, at 5 mice per group, and grouped averages +/- SEM are shown. (PPTX) [file ppat.1006072.s001.pptx]

## Slide 1
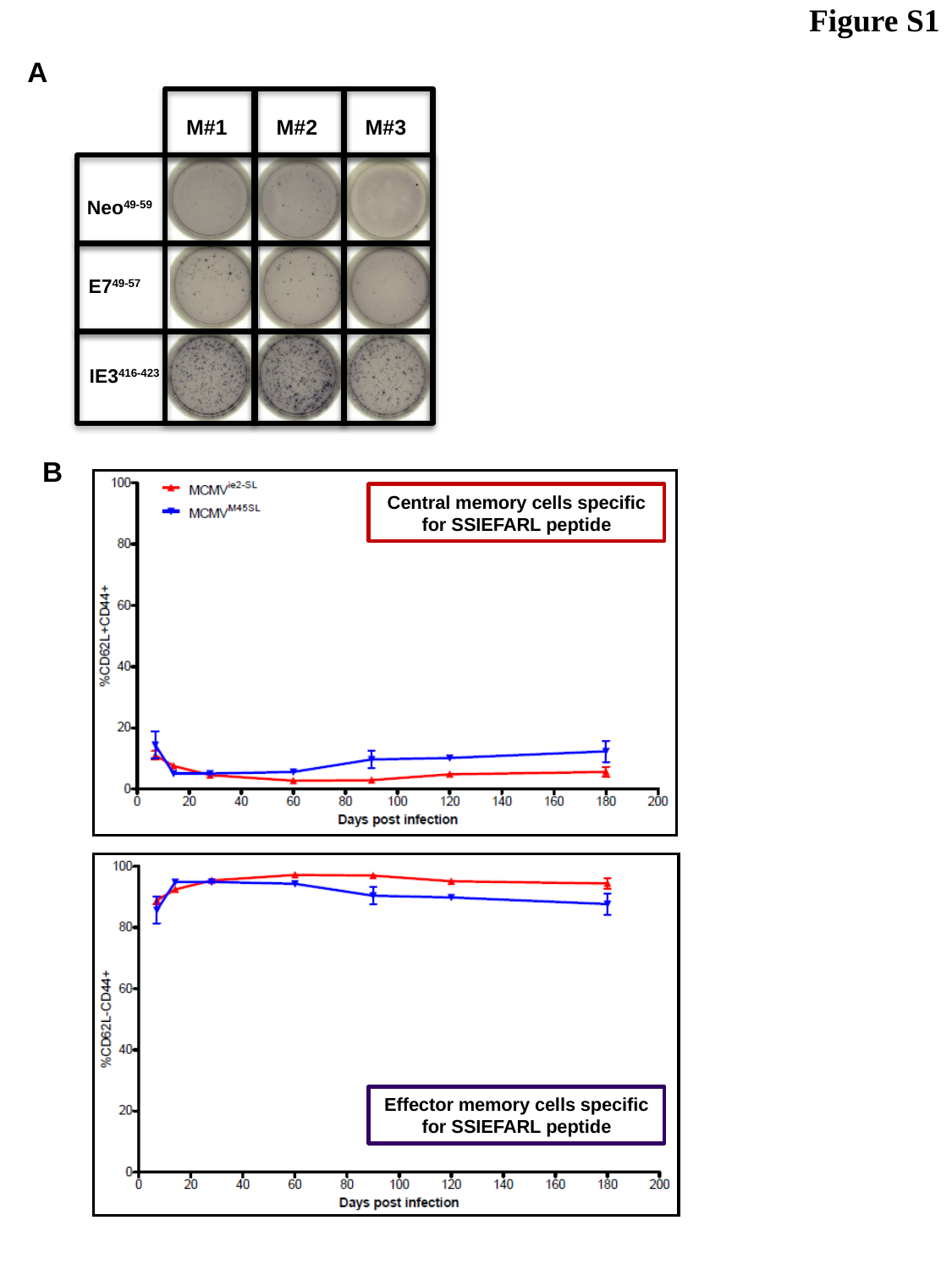

Figure S1
A
M#1
M#2
M#3
Neo49-59
E749-57
IE3416-423
B
Central memory cells specific for SSIEFARL peptide
Effector memory cells specific for SSIEFARL peptide
