# Supplementary material for: Peptide Processing Is Critical for T-Cell Memory Inflation and May Be Optimized to Improve Immune Protection by CMV-Based Vaccine Vectors
Source: PLoS Pathog. 2016 Dec 15;12(12):e1006072. doi: 10.1371/journal.ppat.1006072 (PMC5158087; doi:10.1371/journal.ppat.1006072)
Supplement: S2 Fig — (A) In order to prevent MHC class I presentation of the endogenous HGIRNASFI epitope, its anchoring amino acid (isoleucine) was swapped with the irrelevant amino acid (alanine), which cannot efficiently interact with the peptide-binding cleft of the MHC class I molecule. This resulted in generation of the MCMVM45I->A mutant. (B) A construct AAHGIRNASFI was inserted by means of traceless BAC mutagenesis at the very end of the M45 gene of MCMVM45I->A recombinant (the DNA nucleotide sequence (black letters) as well as the corresponding amino acid sequence (grey letters) are shown). (C) In vitro growth kinetic of MCMVM45I->A and MCMVM45Cterm on NIH3T3 cells. A monolayer of NIH3T3 cells was infected in three independent experiments with indicated viruses at an MOI of 0.1. Medians at indicated time points post infection are shown, vertical bars show standard deviations. (D) Swapping of amino acids in the immunodominant M45Db-restricted peptide and insertion of the peptide in the C-terminus of the M45 protein does not influence viral growth in vivo. 129/Sv mice were i.p. infected with 2x10^5^ PFU of indicated virus. Spleen, liver and lung homogenates were assayed for infectious MCMV titer at day 5 p.i‥ Salivary gland homogenates were assayed at 21 day p.i‥ Each symbol represents one mouse, horizontal lines indicate medians. (PPTX) [file ppat.1006072.s002.pptx]

## Slide 1
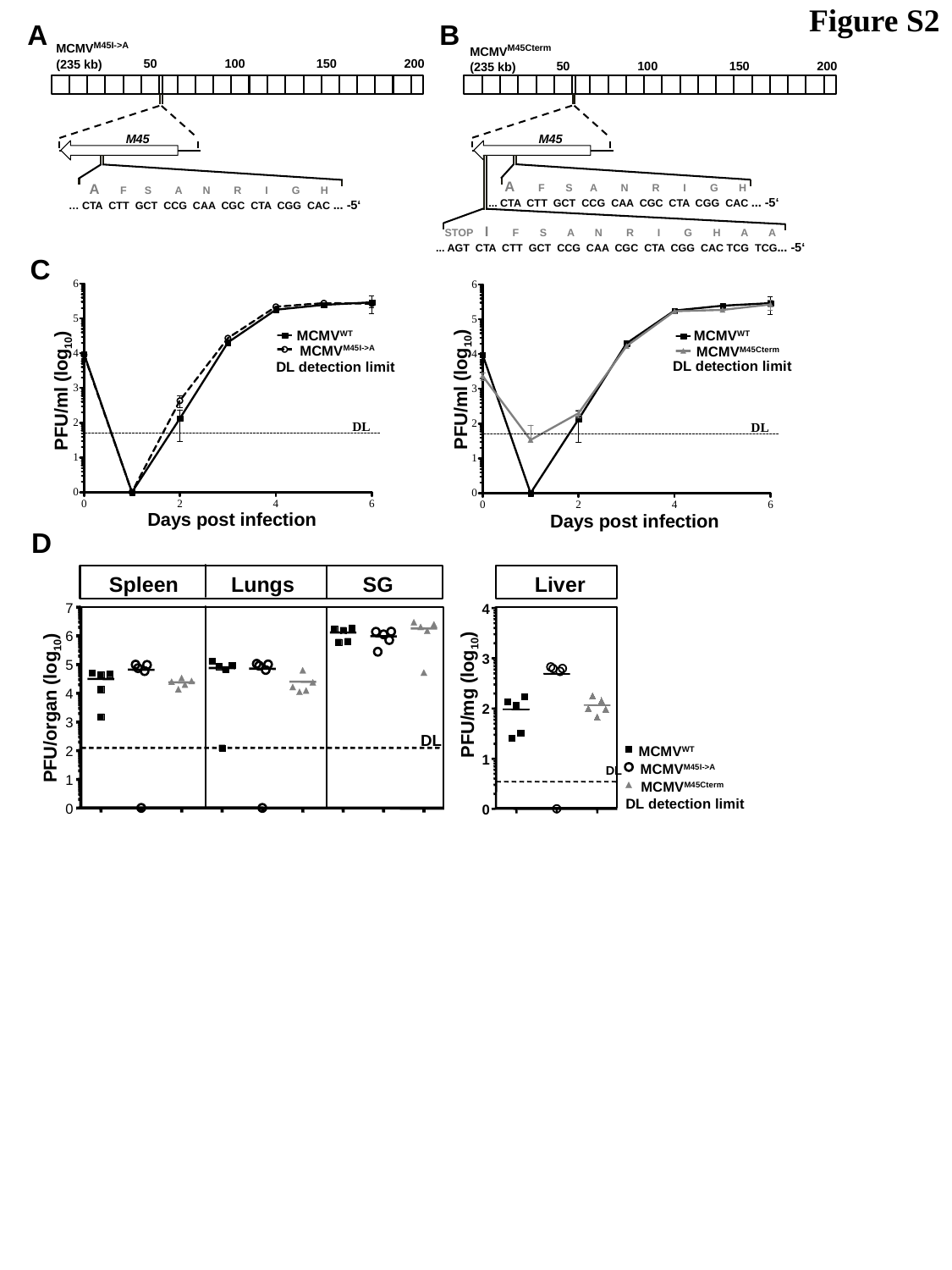

Figure S2
A
B
MCMVM45I->A
(235 kb)
50
100
150
200
M45
 A F S A N R I G H
… CTA CTT GCT CCG CAA CGC CTA CGG CAC ... -5‘
MCMVM45Cterm
(235 kb)
50
100
150
200
M45
 A F S A N R I G H
... CTA CTT GCT CCG CAA CGC CTA CGG CAC ... -5‘
 STOP I F S A N R I G H A A
... AGT CTA CTT GCT CCG CAA CGC CTA CGG CAC TCG TCG... -5‘
C
MCMVWT
MCMVWT
MCMVM45I->A
MCMVM45Cterm
DL detection limit
DL detection limit
PFU/ml (log10)
PFU/ml (log10)
Days post infection
Days post infection
D
Spleen
Lungs
SG
Liver
7
6
5
4
PFU/organ (log10)
3
DL
2
1
0
4
3
2
1
DL
0
PFU/mg (log10)
MCMVWT
MCMVM45I->A
MCMVM45Cterm
DL detection limit
