# Supplementary material for: Peptide Processing Is Critical for T-Cell Memory Inflation and May Be Optimized to Improve Immune Protection by CMV-Based Vaccine Vectors
Source: PLoS Pathog. 2016 Dec 15;12(12):e1006072. doi: 10.1371/journal.ppat.1006072 (PMC5158087; doi:10.1371/journal.ppat.1006072)
Supplement: S3 Fig — (A) Db expression represented as geometric mean fluorescent intensity (GMFI) of the corresponding Ab signal for the experiment shown in Fig 5A. The experiment was performed twice in triplicates and histograms represent the geometric mean values of all data. Error bars show the SEM. (B) Group averages of IFNγ responding cells shown as representative dot blots in Fig 5B. Error bars show the SEM, “+” or “–” indicate respectively whether virus was UV inactivated prior infection or not. (C) MS identity confirmation of target peptide HGIRNASFI. The MS3 spectrum for fragment a82+ (507.78/428.23) of the synthetic reference peptide HGIRNASFI (lower panel) matches the MS3 spectrum of the peptide identified in the IP sample (upper panel) of MCMVM45Cterm infected cells (eluting at app. 18.5 min, see Fig 5C). MS3 spectra were detected for all four fragments, confirming the presence of the peptide HGIRNASFI in the IP sample. Only one fragment MS3 spectrum is shown for clarity reasons. (D) Detailed representation of the M45-specific CD8 T-cell response on day 7 p.i. for the experiment shown in Fig 5D. Each symbol represents a mouse, horizontal lines denote group means. Significance was assessed by Mann-Whitney test. ****—p<0.0001, ns—not significant. (E) Bone marrow chimera mice were generated as described in material and methods section. On day 70 upon bone marrow transfer mice were bled via retro orbital route and percentage of CD45.2+ CD8 T cells (donor cells) in the blood samples was identified by flow cytometry as a marker of chimerism. (PPTX) [file ppat.1006072.s003.pptx]

## Slide 1
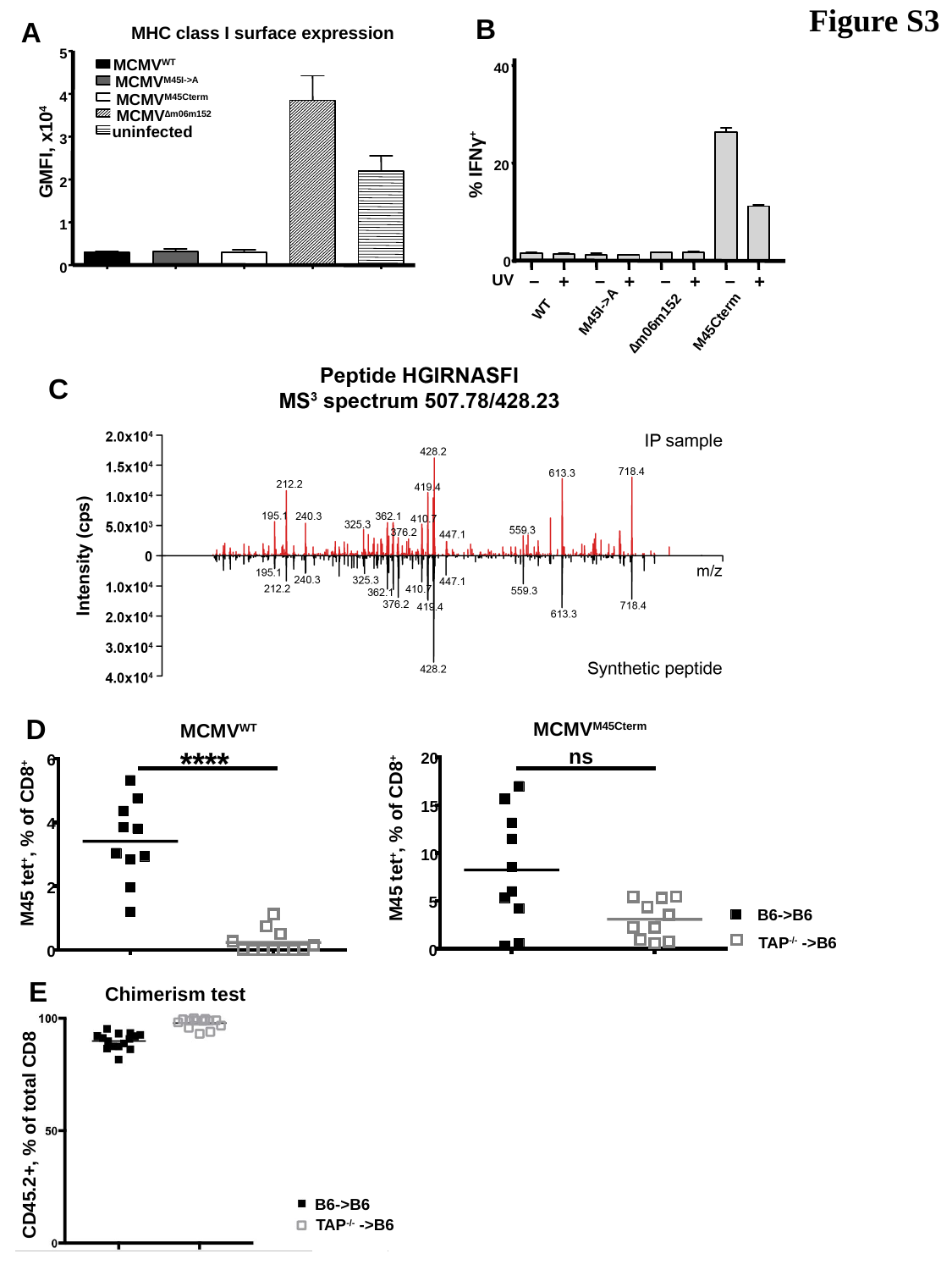

Figure S3
B
A
MHC class I surface expression
5
Legend
Legend
4
Legend
Legend
Legend
3
GMFI, x104
2
1
0
MCMVWT
MCMVM45I->A
MCMVM45Cterm
MCMV∆m06m152
uninfected
40
% IFNγ+
20
0
–
–
–
–
UV
+
+
+
+
WT
M45I->A
∆m06m152
M45Cterm
C
MCMVM45Cterm
MCMVWT
ns
****
20
6
15
4
M45 tet+, % of CD8+
M45 tet+, % of CD8+
10
2
5
B6->B6
TAP-/- ->B6
0
0
D
E
Chimerism test
CD45.2+, % of total CD8
B6->B6
TAP-/- ->B6
