# Supplementary material for: Peptide Processing Is Critical for T-Cell Memory Inflation and May Be Optimized to Improve Immune Protection by CMV-Based Vaccine Vectors
Source: PLoS Pathog. 2016 Dec 15;12(12):e1006072. doi: 10.1371/journal.ppat.1006072 (PMC5158087; doi:10.1371/journal.ppat.1006072)
Supplement: S4 Fig — (A) The MCMV genome area between kilobases 58–59 corresponds to the MCMV gene M45 (enlarged). A construct AASSIEFARL or SSIEFARL was inserted by means of traceless BAC mutagenesis at the very end of the M45 gene of MCMVWT (the DNA nucleotide sequence (black letters) as well as the corresponding amino acid sequence (grey letters) are shown). (B) Growth fitness of MCMVM45ASL mutant compared to MCMVWT. Left graph: C57BL/6 mice were i.p. infected with 106 PFU of indicated virus. Spleen homogenates were assayed for infectious MCMV titer at day 5 p.i‥ Each symbol represents one mouse; horizontal lines indicate medians. Right graph: in vitro growth kinetic of MCMVM45ASL on NIH3T3 cells. A monolayer of NIH3T3 cells was infected in three independent experiments with indicated viruses at an MOI of 0.1. Medians at indicated time points post infection are shown, vertical bars show standard deviations. (C) LSECs were infected with indicated viruses at an MOI of 0.2 with centrifugal enhancement. Splenocytes obtained from gBT-I.1 mice were used as effector cells at an E:T ratio of 3:1. Splenocytes were not restimulated upon isolation from the mice and used untouched for the assay. Co-culture was performed overnight (15h). Columns represent the mean percentage of IFNγ+ cells from 3 independent experiments, and error bars show the SEM. (D) SSIEFARL-specific CD8 T cells (IFNγ+ secreting) from experiment shown in Fig 6B were analysed for the surface expression of CD127 and KLRG1. The staining was used to define the CM (CD127+KLRG1-) and the EM (CD127-KLRG1+) subsets. Grouped means +/- SEM of the percentage of EM (upper graph) or CM (lower graph) cells in the SSIEFARL-responding subset at indicated time points p.i‥ Significance on day 180 p.i. was assessed by Kruskal-Wallis test followed by Dunns post-analysis for MCMVM45SL and MCMVM45ASL infected mice (ns—not significant). (E) Treatment with proteasomal inhibitors does not impair CTL recognition of HGIRNASFI peptide-pulsed target cell [file ppat.1006072.s004.pptx]

## Slide 1
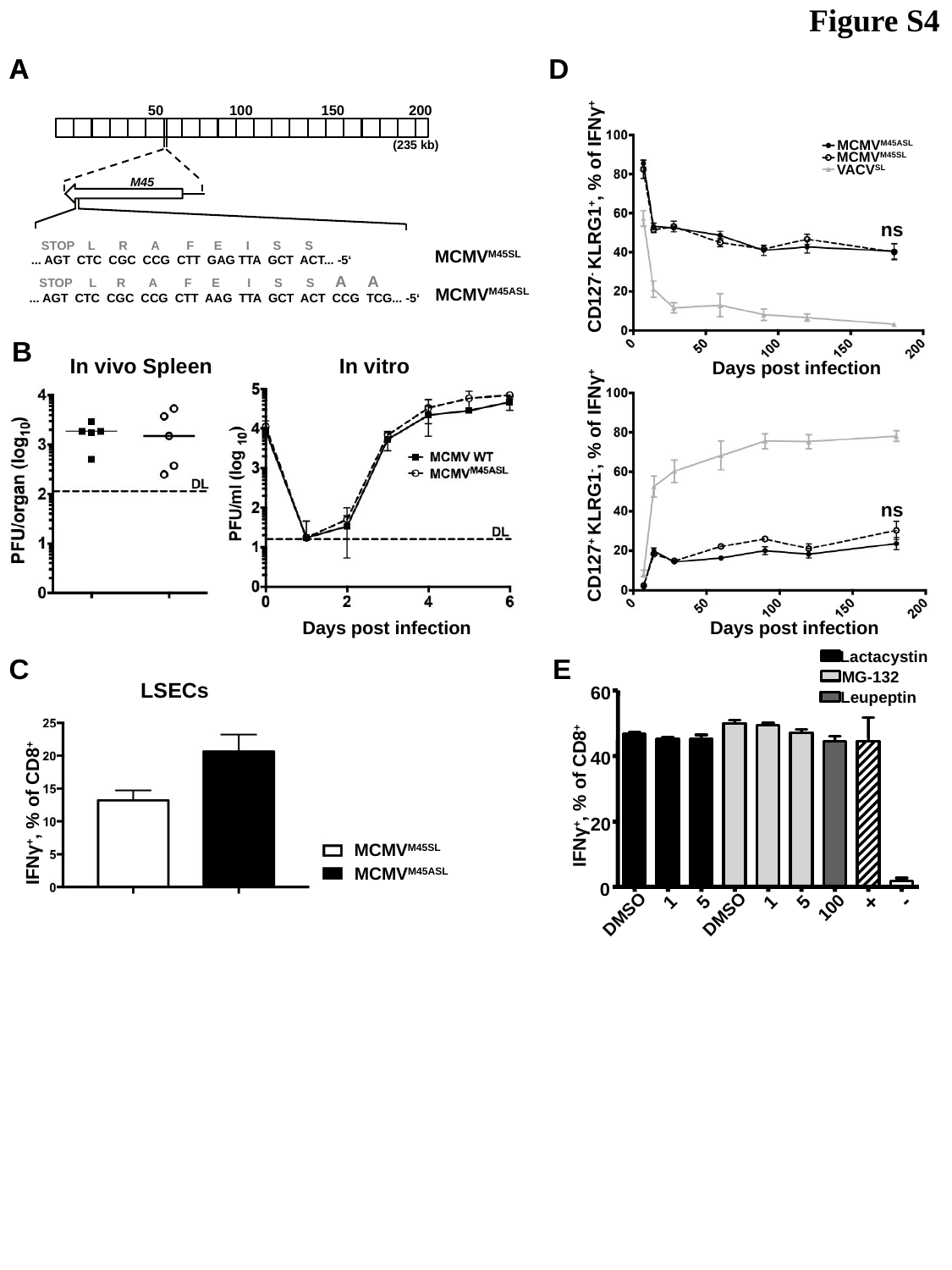

Figure S4
A
A
D
MCMVM45ASL
MCMVM45SL
VACVSL
CD127- KLRG1+, % of IFNγ+
ns
Days post infection
CD127+ KLRG1-, % of IFNγ+
ns
Days post infection
50
100
150
200
(235 kb)
M45
 STOP L R A F E I S S
... AGT CTC CGC CCG CTT GAG TTA GCT ACT... -5‘
MCMVM45SL
 STOP L R A F E I S S A A
... AGT CTC CGC CCG CTT AAG TTA GCT ACT CCG TCG... -5‘
MCMVM45ASL
B
In vivo Spleen
In vitro
Days post infection
C
E
Lactacystin
MG-132
60
Leupeptin
40
IFNγ+, % of CD8+
20
0
-
+
1
5
1
5
100
DMSO
DMSO
LSECs
IFNγ+, % of CD8+
MCMVM45SL
MCMVM45ASL
