# Supplementary material for: Peptide Processing Is Critical for T-Cell Memory Inflation and May Be Optimized to Improve Immune Protection by CMV-Based Vaccine Vectors
Source: PLoS Pathog. 2016 Dec 15;12(12):e1006072. doi: 10.1371/journal.ppat.1006072 (PMC5158087; doi:10.1371/journal.ppat.1006072)
Supplement: S1 Table — (DOCX) [file ppat.1006072.s005.docx]

**Supplementary Table 1** *List of Primers used in the study*

| Name | Sequence | Template |
| --- | --- | --- |
| **Primers used for generation of pGP704SceIKn** | | |
| SceIKn fwd | caagaattctagggataacagggtaatcgatttcgatttattcaacaaagccacgt | pGP704-Kan_2 FRT-Kan-FRT |
| SceIKn rev | Gttgaattcctccggagccagtgttacaaccaattaaccaattctgatt |  |
| **Primers used for generation of MCMV^M45ASL^** | | |
| M45ASL new fwd | aagcccactcgagcgccagagcaatagaactcgttttttggcgacgagttcgccgtcagaggcgggcgaattcaatcgatgaggcagctagggataacagggtaatcgat | pGP704SceIKn |
| M45ASL int rev | GCCCCCTTCCGTGAATTATCGCGCTGCCTCATCGATTGAATTCGCCCGCCTCTGACGGCGAACTCGTCGCCAGTGTTACAACCAATTAACCAATT |  |
| M45ASL new fwd | Aagcccactcgagcgccagagcaatagaactcgttttttg | PCR product from reaction with 2 previous primers |
| M45ASL ext rev | TGTCCGTCGGAGTATATAAATGTAGTATGCCCCCTTCCGTGAATTATCGC |  |
| **Primers used for swapping anchoring isoleucin to alanine in the HGIRNASFI peptide** | | |
| M45 I to A int Fwd | gttggcggcttcgtcggccgagcagccggttgcgaaggaggcgttcctgatgccgtgtagggataacagggtaatcgatt | pGP704SceIKn |
| M45 I to A int Rev | TGAAGACGTGGTGAAACACGGCATCAGGAACGCCTCCTTCGCAACCGGCTGCTCGGCCGACGAGTGTTACAACCAATTAACCAATTCTGA |  |
| M45 I to A elong Fwd | cccggagtggtgcccgccaggttggcggcttcgtcggccg | PCR product from reaction with 2 previous primers |
| M45 I to A elong Rev | TTCCTCGAGAAACTACGTGAAGACGTGGTGAAACACGGCAT |  |
| **Primers used for insertion of the HGIRNASFI peptide in the C-terminal end of the M45 protein** | | |
| AAdM45 int fwd | Tcgttttttggcgacgagttcgccgtcagatgaacgaggcgttgcggatgccgtgagcagcgcgataattctagggataacagggtaatcgatttcgatt | pGP704SceIKn |
