# Supplementary material for: Peptide Processing Is Critical for T-Cell Memory Inflation and May Be Optimized to Improve Immune Protection by CMV-Based Vaccine Vectors
Source: PLoS Pathog. 2016 Dec 15;12(12):e1006072. doi: 10.1371/journal.ppat.1006072 (PMC5158087; doi:10.1371/journal.ppat.1006072)
Supplement: S2 Table — (DOCX) [file ppat.1006072.s006.docx]

**Supplementary Table 2** *List of recombinant MCMVs used in the study*

| **Virus name** | **Description** |
| --- | --- |
| MCMV^E6+E7^ | Expresses the full-length E6 and E7 proteins of the HPV16 under the control of the HCMV IE promoter, because we showed previously that an IE promoter. Lacks the viral genes *m1* to *m16* thus providing ample cloning capacity. Contains E7_49-57_ epitope at its native position |
| MCMV^ie2E6-7full^ | Full-length E6 and E7 proteins are fused to the C-terminus of ie2 protein. Contains E7_49-57_ epitope at its native position |
| MCMV^ie2E7^ | Expresses E7_49-57_ epitope from the C-terminal end of ie2 protein. |
| MCMV^∆m06m152^ | In this virus immune evasion genes *m06* and *m152* were deleted resulting in absence of surface presentation of peptide-MHC-I molecules |
| MCMV^ie2SL^ | HSV gB derived epitope (SSIEFARL) is inserted at the C-terminal end of ie2 protein |
| MCMV^M45SL^ | HSV gB derived epitope (SSIEFARL) is inserted at the C-terminal end of M45 protein |
| MCMV^M45ASL^ | HSV gB derived epitope (SSIEFARL) is inserted at the C-terminal end of M45 protein. Additionally 2 Alanines were introduces at the N-terminus of the epitope - AASSIEFARL |
| MCMV^M45I->A^ | C-terminal Isoleucine of the M45 D^b^ epitope (HGIRNASFI) was swapped to Alanine that preventing epitope binding to MHC-I and its subsequent surface presentation |
| MCMV^M45Cterm^ | Was generated on the backbone of MCMV^M45I->A^ mutant. In this virus HGIRNASFI epitope was inserted at C-terminus of M45 protein resulting in its presentation exclusively from C-terminus. |
